# Supplementary material for: A network-centric approach for estimating trust between open source software developers
Source: PLoS One. 2019 Dec 30;14(12):e0226281. doi: 10.1371/journal.pone.0226281 (PMC6936801; doi:10.1371/journal.pone.0226281)
Supplement: Attachment — Submitted filename: Response to Reviewers.pdf [file pone.0226281.s001.pdf]

Rebuttal Letter

Thanks to the editor and the reviewers for very helpful comments.

We have addressed all the comments in the revision. For each comment, we identify the page number of the manuscript on which we made the pertinent changes; on the specified page of the manuscript with tracked changes, we have inserted an arrow in the margin (with the specified tag) that points approximately to the changed text.

Note: We tried *latexdiff* to track changes but the tex file produced by *latexdiff* did not compile. Thus, we used the approach with markers in the margin to track changes.

Responses to Review Comments

E1. In your Methods section, please include additional information about your dataset and ensure that you have included a statement specifying whether the collection method complied with the terms and conditions for the website.

Response: We describe our data collection and preprocessing methods in Section 4.1. We use official GitHub API for collecting the data. We included a statement indicating that our data collection method complies with GitHub's API terms (Tag E1 on Page 11).

C1. In general, there are a few spots in which some conclusions are drawn that, I'd argue, are not fully supported by the data; an example is outlined below (line 428). As the primary consideration is experimental robustness and not "practical applicability", I would suggest toning back some of the stronger results statements. Most statements are acceptable and the subtlety is appreciated (e.g., lines 407-412 and lines 418-419).

Line 428: The statement that Fig 4 provides"strong evidence" for the given conclusion is a stretch; Cliff's delta is either small or negligible in all cases. Though journal guidelines dictate that research "impact" is not a considered guideline for publication, this statement of "strong evidence" is a stretch. I'd suggest removing the "strong" adjective here.

Response: We updated the conclusion drawn on line 428 (now, line 531). We removed "strong" and rephrased the sentence (Tag C1 on Page 18).

C2. Line 43: How is a "top" Python project defined?

Response: These were "top" projects based on the number of pull requests. However, we felt that the word "top" can be misleading. So, we removed "top" but clarified how these 179 projects were selected (Tag C2 on Page 11).

C3. Line 92–95: This description is somewhat confusing. How does each parameter take a value in [0, 1], yet three parameters add up to 1? I believe this is better explained later in the paper, but some small introduction or explanation here would make things a bit easier to follow.

Response: We updated the description in lines 92–95, clarifying that the value one trust parameter can take is constrained by the values of the other two trust parameters (Tag C3 on Page 3). We also indicate two special scenarios when an opinion is considered dogmatic or vacuous.

C4. Line 209–210: To my knowledge, there is no precedent that the mean of word vectors in a document is representative of the content as a whole; only that word

vectors are pair-wise close to each other in embedding space as measured by cosine similarity (and in compositions of cosine similarity). I would suggest citing a reference to such work, or explicitly stating this as a threat to validity.

Response: We agree that a simple word vector averaging technique for representing a sentence may not yield an optimal representation of the sentence as a whole. We include a Threat to Validity, acknowledging this. Further, we note that the simple averaging techniques has been successful in some of the existing applications (Tag C4 on Page 20).

C5. Section 3.2.3: It is unclear at what level the observations (i.e., "rows") are for the regression. Are they per-comment? Per pull request? Later (in results) this becomes clear, but stating it here may be a good idea.

Response: We employ each interaction between a pull request evaluator and generator, within one pull request, as an observation in the regression model. We clarified this (Tag C5.2 on Page 7). We also clarify that a pull request can contain more than one interaction and an interaction can contain more than one comment (Tag C5.1 on Page 7).

C6. Fig. 2 / Section 3.2.3: What type of cross-validation was performed? K-fold (with what value of k)?

Response: We repeat each opinion prediction experiment 30 times. In each repetition, we randomly split the data in 7:3 ratio for training and testing, respectively. We describe this in Section 4.2.1 (Tag C6.1 on Page 12). We perform 30 repetitions as opposed to K-fold cross-validation (say, with K=10) so that we get more observations, which gives us a better sense of the variance in the results.

- Would results change if cross-validation was performed with respect to time? Contribution guidelines and the expected quality of accepted pull requests may change drastically over time, and may affect underlying trust relationships.
  Response: We added an experiment for time-based validation. Section 4.2.1 describes the experiment (Tag C6.2 on Page 13) and Table 5 shows the results (Tag C6.3 on Page 15).
- Would results change if cross-validation was performed across repositories? Projects have different views of acceptable pull request quality / submission behavior, which will likely affect trust relationships.
  Response: We agree that standards of opinions may vary across repositories. However, performing a validation specific to repositories is challenging with our dataset since we only have a few interactions from each repository (just one or two for most repositories). We included this as a threat to the validity of our results under generalizability (Tag C6.4 on Page 20).

C7. Line 329-331:

- Re (2): How are auto-generated comments detected?
  Response: We remove a specific type of autogenerated comments—those about code coverage, which have a specific string at the beginning of the comment body (Tag C7.1 on Page 12).
- Re (4): What precisely constitutes "referring to the pull requester"? The usage of an @-mention directed towards the pull requester? Or some other signaling mechanism?
  Response: Yes, we used the @-mentions to detect comments directed toward the pull request generator (Tag C7.2 on Page 12).

C8. Line 333: How was this replacement performed? Is a list of mappings between abbreviations and their full forms available?

Response: We manually created a list of abbreviations that occurred in our annotated interactions. The list is not extensive since our annotated dataset was small. We explicitly mention these abbreviations now (Tag C8 on Page 12).

C9. Section 4.2 / 5.1: Interpreting performance based on MAE without a clear baseline is difficult. What is the MAE in the worst case scenario? E.g., when always assigning a 5 when the true value is a 1, and analogously assigning maximum "worst possible values" to the test data. What would the MAE be if one, e.g., randomly assigned labels (uniformly from 1 to 5) to the test set?

Response: We added two baselines—a random classifier and a majority-class classifier—as described in Section 4.2.1 (Tag C9.1 on Page 13). Fig 6 compares our best performing opinion prediction models with the baselines (Tag C9.2 on Page 15). Tables 6 and 7 show the results from statistical significance and effect size tests comparing our models with the baselines (Tag C9.3 on Page 15).

- A table or figure representing the distribution of labels in the train and test set would also be helpful here.

  Response: We added Table 4 showing the distribution of the opinion labels in training and test sets (Tag C9.4 on Page 12).

- In addition, I would also appreciate a variance-based (or at least "variance informed") evaluation metric in addition to MAE.

  Response: We now employ Kruskal-Wallis, and Dunn's multiple comparison tests for comparing the MAE samples (from 30 repetitions) of different opinion prediction techniques and the baselines (Tag C9.5 on Page 13). These tests are variance informed. In addition, instead of showing only the mean MAE, we now show violin plots in Figures 4, 5, and 6, displaying the distribution of MAE values across the repetitions (Tag C9.6 on Page 15).

C10. Was classification, as opposed to regression, also considered here? I understand that the regression approach preserves distance between classes defined on a Likert (or Likert-like) scale, but I am curious as to whether the authors believe a classification approach would drastically change their results.

Response: We added experiments for the classification techniques. Each of the algorithms we employ for regression can also be employed for classification with appropriate settings (Tag C10.1 on Page 7). Fig 4 compares the five regression models and Fig 5 compares five classification techniques for opinion prediction (Tag C10.2 on Page 15). We employed the best performing classification and regression techniques in the experiments for indirect trust computation as shown in Table 8 (Tag C10.3 on Page 16).

C11. Section 4.3 / 5.2.2: I very much appreciate the usage of a simple baseline model for comparison. Is there a reason that you did not include features used in prior work (such as in the cited work by Tsay et al. [14], who build a model for pull request acceptance as done in this work)?

Response: Our notion of trust may already capture several features Tsay et al. include, e.g., social connection and prior interaction. Including such features along with trust in a predictive model may result in complex interactions between features. Such interactions are not easily recognizable, making it hard to assess whether or not trust (as we compute it) is a useful metric to predict pull request acceptance or not. That is why we include a simple baseline to compare our model

against. Further, as we acknowledge in the Threats to Validity, our objective was not to develop the best performing PR-classification model but to demonstrate that a predictive model best on trust can be valuable (Tag C11 on Page 20).

C12. Section 5: As stated above, it is unclear what method of cross-validation is performed. Please clarify.

Response: Similar to opinion prediction experiments, we repeat pull request classification experiments 30 times, too (Tag C12.1 on Page 14). We show violin plots comparing the precision, recall, and F_1 scores of the classification models from the 30 observations (Tag C12.2 on Page 19). We also perform statistical significance and effect size tests (Tag C12.3 on Page 19).

C13. Table 5: I assume this performance is calculated on the test set, but an explicit statement of such in the table caption would be useful.

Response: This evaluation is based on 1% ground truth edges selected from D_{train} as described in Section 4.2.2 (Tag C13 on Page 13). We also updated the caption of Table 8 (previously Table 5) to reflect this.

C14. Line 381–388: It is stated that trust is computed in the CDN described by D_{train}. Is this trust value then considered "fixed" and used in the corresponding models trained on 70% of data from D_{test}? A bit of additional explicit description and clarification could be added here.

Response: Yes, the trust values we compute in D_{train} are considered fixed. However, we need to compute a trust value for each pull request by combining the trust values from the members of a project (to which the pull request was submitted) to the pull request generator. We clarified this (Tag C14 on Page 14).

C16. Fig 5: This plot is hard to read (both in image quality and presentation). The addition of a contour plot, estimated 3-d surface, or some other positional indicator(s) (e.g., a line drawn from the horizontal plane vertically to each point on the vertical axis) would greatly increase readability.

Response: We redrew this figure (now Fig 8) with much better resolution (Tag C16 on Page 18). Further, we color coded each region of trust to make the comparison of the number of points in different regions of trust easier. This plot would be easier to read if it is interpreted in conjunction with Table 9.

C17. Section 5.2.1 / Table 6: What part of the data are these values drawn from? The 30% of D_{test} described in Section 4.3? Table 3 states that there are 13,765 pull requests in the test set, but there are 3,610 data points in each cell of Table 6.

Response: Table 6 is now Table 9. It contains 1805 accepted and 1805 rejected pull requests. We clarified how we arrived at these many pull requests in Section 4.3.1 (Tag C17 on Page 14).

C18. Grammar / Typos:

Response: Fixed.
